# Orthodontic management by functional activator treatment: a case report

**DOI:** 10.1186/s13256-017-1505-y

**Published:** 2017-12-02

**Authors:** Giuseppe Aprile, Eleonora Ortu, Ruggero Cattaneo, Davide Pietropaoli, Mario Giannoni, Annalisa Monaco

**Affiliations:** 1 MeSVA Department, Division of Dentistry, University of L’Aquila, P. le Salvatore Tommasi, 67100 L’Aquila, Italy; 2 Giuseppe Aprile, Freelance Doctor of Dental Surgery (DDS), Rome, Italy

**Keywords:** Overjet, Malocclusion, Teeth, IOTN, Activator

## Abstract

**Background:**

Managing orthodontic treatment is often very difficult for the orthodontist. Many devices are used during the orthopedic phase of orthodontic treatment, always with different functions. We describe a case of orthodontic management treated with the Equilibrator O.S.A. device (equilibrator designed by Ovidi, Santi, and Aprile for Eptamed SRL; Cesena, Italy; www.eptamed.com).

**Case presentation:**

A healthy 10-year-old white boy presented with a skeletal class II, division 1 malocclusion, molar class II, exhibiting an overjet of 7 mm prior to treatment. For treatment, we only used the Equilibrator O.S.A. device.

**Conclusions:**

We successfully treated an orthopedic/orthodontic case with a particular device that we describe here.

## Background

Functional activators were created for orthodontic purposes in the 1950s by Soulet and Besombes, two French orthodontists. These functional orthopedic devices were designed to reinstate the craniofacial architecture. The first Soulet-Besombes appliances were made of natural rubber because its elasticity was able to produce a well-controlled orthopedic effect. This technique was defined by the French orthodontic inventors as a way to free, stimulate, and lead the growth of jaws [1–4]. In subsequent years, pursuant to the Soulet and Besombes orthodontic philosophy, many functional activators were developed, with several shapes, different materials, and various orthodontic purposes. Activators balance the skeletal bases through two double matched planes, upper and lower, where teeth are positioned with effects of propulsion, retropulsion, and expansion. After employing the positioners, the orthodontist will require minor tooth movement after functional treatment because of the elastomeric material [5, 6]. In fact, this device improves the chewing function, aligns the teeth, re-educates the tongue due to stimulation toward the retroincisal papilla spot, and modulates the muscular tone in occlusal-postural syndrome; moreover, it is ideal for treating obstructive sleep apnea syndrome. The employed materials are suitable because they are soft enough to allow patient compliance without traumatizing the oral mucosa and jaws and at the same time are tough enough to resist chewing loads. There is a complete array of activators for every type of mouth, according to the skull conformation, body features, and dental arch shape. Proper employment of this activator in association with physical exercises will allow patients to obtain benefits in the entire neuromyofascial system [7–11]. The aim of this report is to describe a patient with a class II, skeletal I division, molar class II mandibular deficit, who exhibited both overjet and overbite between 6 and 9 mm prior to treatment. This case was solved during mixed dentition by using only the activator Equilibrator (EQ) O.S.A. ideated by Doctors Ovidi, Aprile, and Santi and commercialized by Eptamed (EQ O.S.A.; Eptamed SRL, Cesena, Italy; www.eptamed.com) according to orthopedic-functional orthodontics.

## Case presentation

**Fig. 1:**
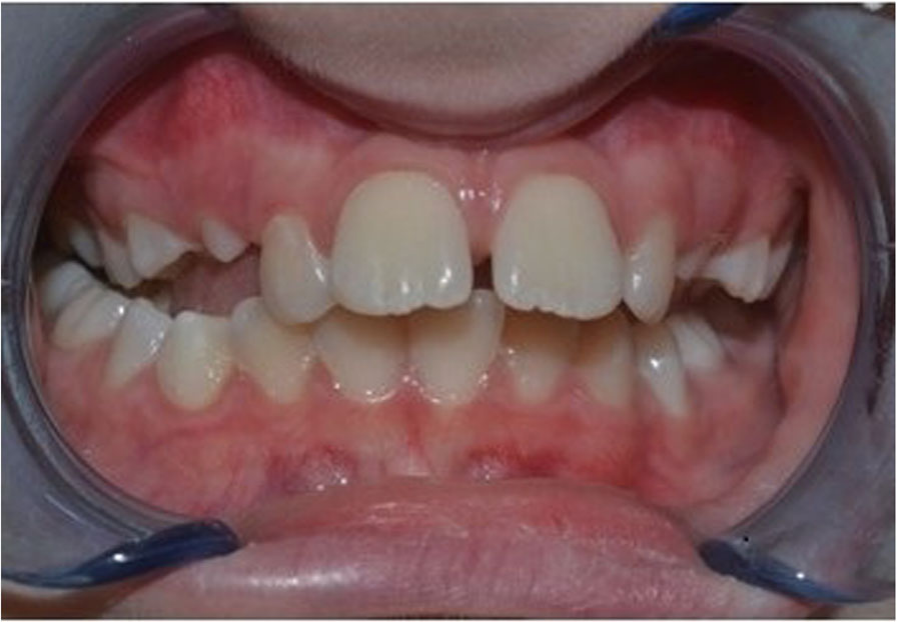
Intraoral photographs before starting the orthopedic-functional orthodontic therapy

**Fig. 2:**
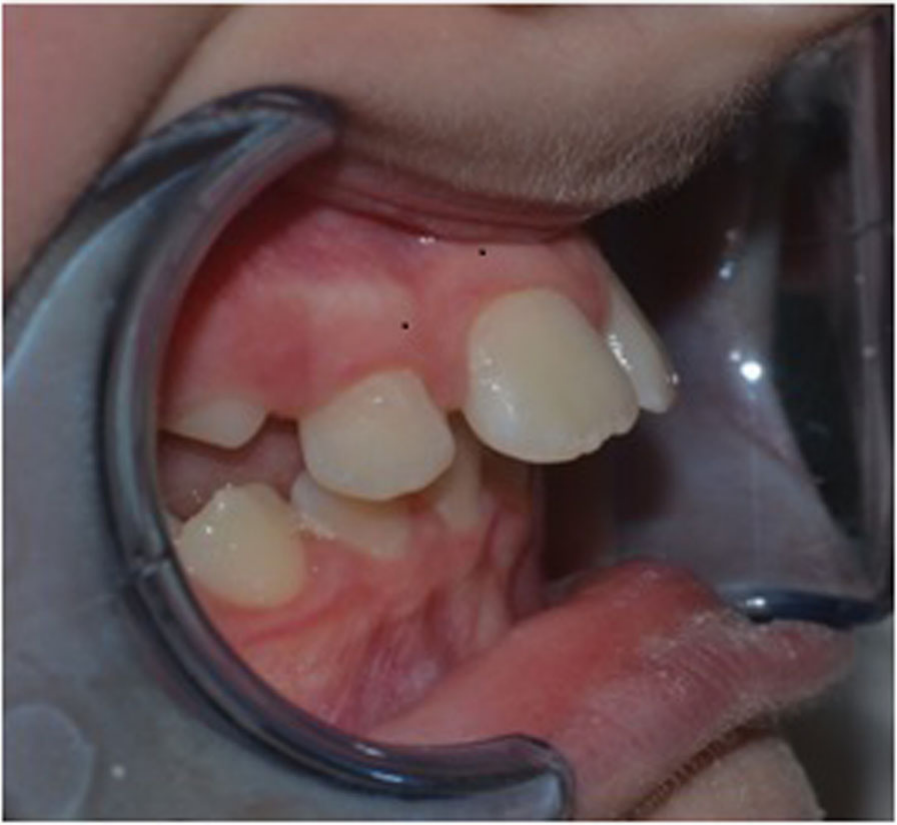
Intraoral photographs before starting the orthopedic-functional orthodontic therapy

**Fig. 3:**
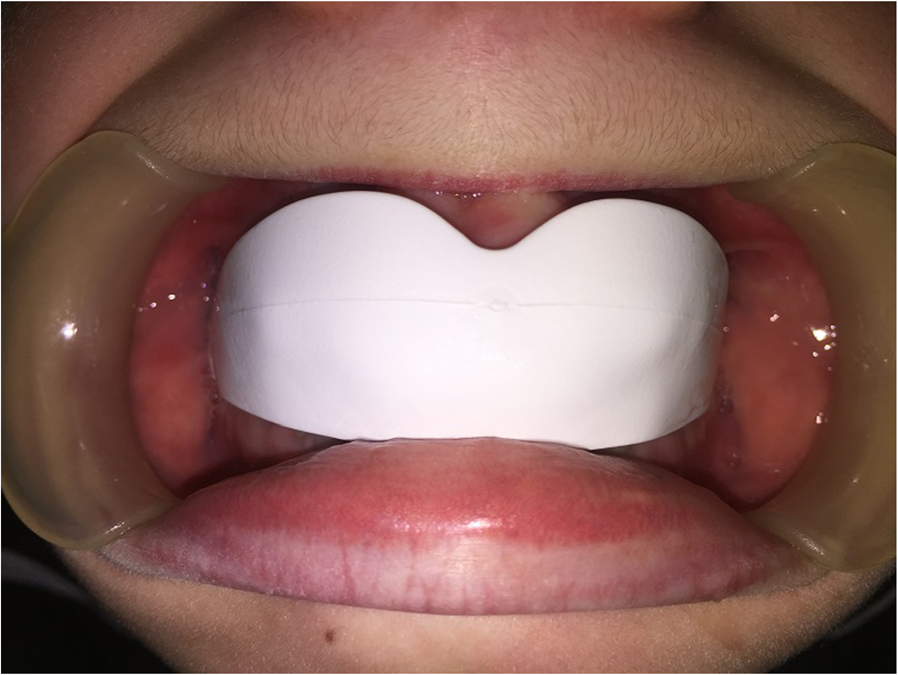
The Equilibrator O.S.A. device in the oral cavity of the patient

This study was conducted in accordance with the fundamental principles of the Declaration of Helsinki. A healthy 10-year-old white boy (without any types of diseases) was clinically examined at the Dental Clinic of the University of L’Aquila. He was examined by the same clinician (AM) who prescribed a dental panoramic radiograph, acquired extraoral and intraoral photos, and took alginate impressions of both dental arches. Based on these data, the orthodontist created a treatment plan specific to the patient according to the Index of Orthodontic Treatment Need (IOTN) described by Brook and Shaw [12]. The diagnosis was a skeletal class II, division 1 malocclusion, molar class II, exhibiting both an overjet of 7 mm prior to treatment. According to the IOTN, our patient displayed a grade 4 treatment requirement (increased overjet > 6 mm but ≤ 9 mm; extreme lateral or anterior open bites > 4 mm). He showed a medium grade of dental crowding in both dental arches, interincisive diastemata between the upper central incisors, and an increased overjet because of tongue interposition (Figs. 1 and 2). Fortunately, he did not have any speaking or eating problems. His dental and oral health was good; in fact, he was also under the care of the dental hygienist of our Clinic. He was treated only with the use of the EQ O.S.A. 4 device (white, in natural rubber) that will be described subsequently (Fig. 3), which was replaced two times after 6 months, during 1 year of orthopedic-functional orthodontic therapy. He was under treatment until full permanent dentition was completed.

### EQ O.S.A. device

The EQ O.S.A. (Eptamed-produced) is the result of a coordinated effort among eminent chemistry and technology manufacturers to provide orthodontists a valid aid to optimize clinical outcomes. It shows a wide therapeutic range. The shape was defined in 2005 and since then it has been used in many clinical cases with excellent outcomes. This equilibrator yields several benefits: improving the chewing function, aligning the teeth, re-educating the tongue due to stimulation toward the retroincisal papilla spot, and modulating the muscular tone in occlusal-postural syndrome; furthermore, it is ideal for treating obstructive sleep apnea syndrome. After taking alginate impressions and developing cast stone models, the orthodontist uses an appropriate ruler to measure the distance between the palatal cusps of the first upper bicuspids (or the first upper deciduous molars) and will choose the correct size among the following:

- OSA 3 – from 24 to 27 mm in mixed dentition;
- OSA 4 – from 28 to 31 mm in mixed dentition;
- OSA 5 – from 32 to 36 mm in permanent dentition.

**Fig. 4:**
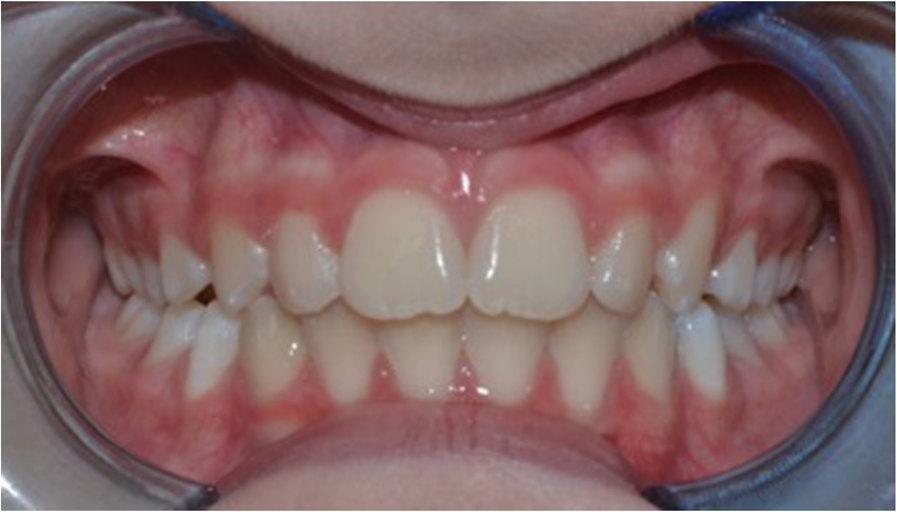
Intraoral photographs at the end of the orthopedic-functional orthodontic therapy using the EQ O.S.A. device

**Fig. 5:**
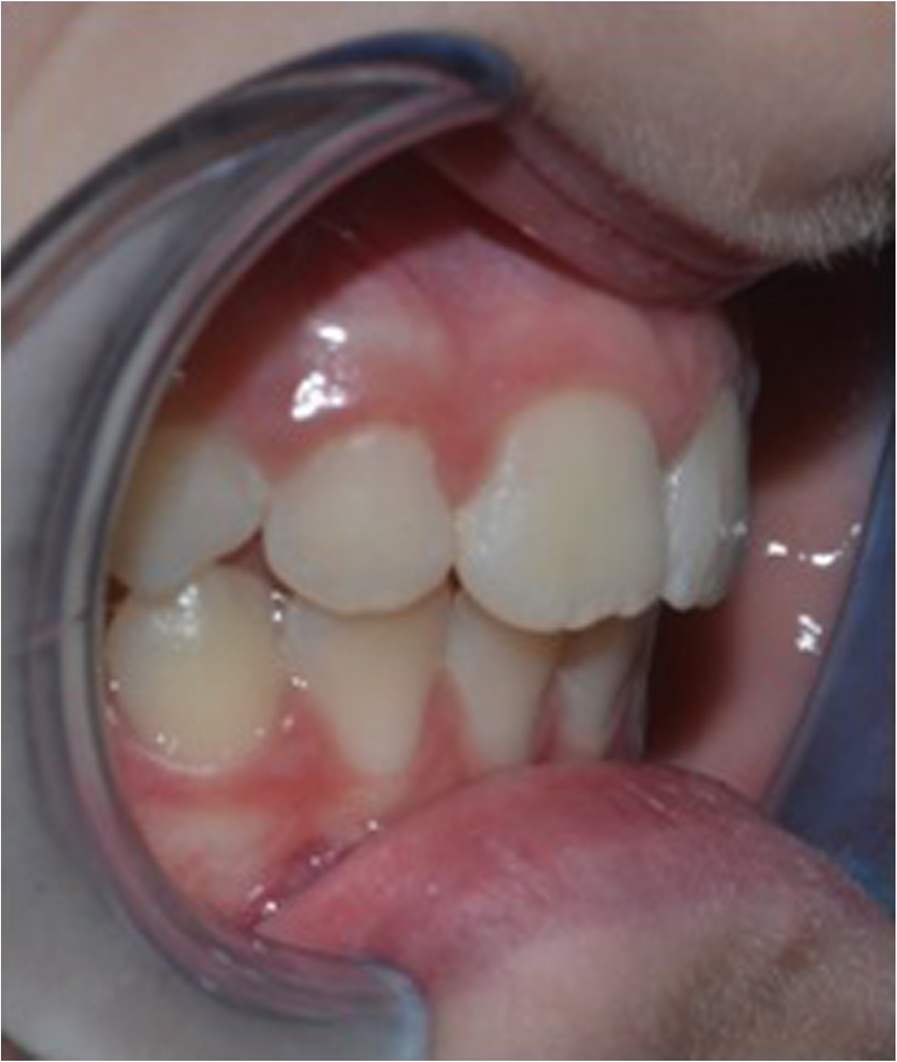
Intraoral photographs at the end of the orthopedic-functional orthodontic therapy using the EQ O.S.A. device

Three different materials are available based on hardness: white in natural rubber (soft), lavender in elastomeric resin (medium), and mint in elastomeric resin (hard). The patient inserts his or her teeth in the fitting upper and lower splints. This device is functionalized by biting it, through soft elastic forces led by muscle energy. The activator is worn all night long and for 1 hour during the day. The activator can be worn in the day while reading, driving, or studying, but not while playing sports. Moreover, two exercises of 15 minutes each are executed, in the morning and in the evening. The basic exercise can be adapted to the patient’s features and characteristics. In Souchard’s frog position, attention is paid to breathing slowly and softly biting the activator during inspiration and releasing the jaws during expiration. The tongue remains at its position. Coordination might be difficult in the beginning, but after a few times, the patients often become familiar with it. The orthodontist checks the patient every 45 days to evaluate eventual modifications for execution on the device. The appliance must be replaced every 6 months, according to the orthodontist’s evaluations, until reaching the final orthodontic outcome. After 1 year of using the EQ O.S.A. device, our patient’s IOTN passed from grade 4 to a grade 1, as shown in Figs. 4 and 5. He completed the orthodontic treatment with a satisfactory alignment of both dental arches, and his tongue function was rehabilitated (final swallowing with the tongue in its position).

## Discussion

Recently, knowledgeable orthodontists intending to solve cases have focused their attention on the etiopathogenesis of malocclusions instead of single clinical features. This orthodontic approach is aimed at maintaining teeth in the corrected positions after treatment and preventing orthodontic relapse. Atypical swallowing is a myofunctional problem, which constitutes a hot topic in orthodontics because it is challenging to treat. Tongue thrusting (an abnormal tongue position deviating from the physiological swallowing pattern) and mouth breathing may be associated with anterior open bite, transverse contraction of the maxillary dental arch, abnormal speech, interincisive diastema, and anterior protrusion of the maxillary incisors. It appears that several factors account for the persistence of infantile swallowing patterns and that tongue thrust plays an important role in the etiology of open bite as well as in the relapse of patients with treated open-bite. A low tongue posture is associated with mouth breathing and the development of inflammatory diseases such as otitis, tonsillitis, sinusitis, and adenoid hypertrophy [13–16]. Last, but not least, mouth breathers with a low tongue posture often have chronic neck pain and poor posture. The literature shows that the tongue and neck muscles are related because of common proprioception by a common trunk from the ansa cervicalis through the hypoglossal nerve [17–19].

The EQ O.S.A. is a type of orthodontic appliance that stimulates growth and, through the input of muscle movements, elicits tissue development toward a suitable chewing function. The device is actually an orthopedic-functional appliance. Biting this elastomeric device balances tension up to the sphenobasilar synchondrosis, according to osteopathic medicine and philosophy. The teeth’s positions are determined by this new skull harmony through an osteopathic effect [20]. Laganà and Cozza have described a similar case treated with a similar device [6]. The EQ O.S.A. device correctly employed allows a healthier posture and freer movements of the tongue. The apex of the tongue touching its spot during swallowing together with the centripetal force of perioral muscles act as an orthodontic device to enhance reduction of the overjet, improvement of tongue function and, thereby, an enhancement of breathing, with functional and esthetic improvements.

## Conclusions

The aim of this case report is to allow the orthodontist reader to learn the basic clinical skills for this appliance, which is easy to use and comfortable to wear. Enhancing the dynamic function, and thereby the cause of the malocclusion, makes orthodontic relapse much less likely and thus maintains stable occlusion over time. Proper use of the activator, in association with exercises and techniques for muscular balance, will allow the patient to gain benefits throughout the neuromyofascial system – with fewer concerns for the orthodontist.

## Abbreviations

EQ O.S.A.: Equilibrator O.S.A.
IOTN: Index of Orthodontic Treatment Need
